# Endotoxin and cytokine reducing properties of the oXiris membrane in patients with septic shock: A randomized crossover double-blind study

**DOI:** 10.1371/journal.pone.0220444

**Published:** 2019-08-01

**Authors:** Marcus E. Broman, Fredrik Hansson, Jean-Louis Vincent, Mikael Bodelsson

**Affiliations:** 1 Lund University, Skåne University Hospital, Department of Clinical Sciences Lund, Anaesthesiology and Intensive Care, Lund, Getingevägen, Lund, Sweden; 2 Clinical Trial Consultants, Uppsala, Dag Hammarskjölds Väg, Uppsala, Sweden; 3 Department of Intensive Care, Erasme University Hospital, Université Libre de Bruxelles, Brussels, Belgium; Universidade do Extremo Sul Catarinense, BRAZIL

## Abstract

**Background:**

Endotoxin induces an inflammatory response, with secondary release of cytokines, which can progress to shock and multiple organ failure. We explored whether continuous renal replacement therapy (CRRT) using a modified membrane (oXiris) capable of adsorption could reduce endotoxin and cytokine levels in septic patients.

**Methods:**

Sixteen patients requiring CRRT for septic shock-associated acute renal failure and who had endotoxin levels >0.03 EU/ml were prospectively randomized in a crossover double-blind design to receive CRRT with an oXiris filter or with a standard filter. Endotoxin and cytokine levels were measured at baseline and 1, 3, 8, 16 and 24 hours after the start of CRRT. Norepinephrine infusion rate and blood lactate levels were monitored.

**Results:**

During the first filter treatment period, endotoxin levels decreased in 7 of 9 (77.8%) oXiris filter patients, but in only 1 of 6 (16.7%) standard filter patients (*P* = 0.02). Levels of tumor necrosis factor (TNF)-α, interleukin (IL)-6, IL-8 and interferon (IFN)γ decreased more with the oXiris filter than with the standard filter. Lactate concentration decreased with oXiris (-1.3[-2.2 to -1.1] mmol/l, *P* = 0.02), but not with the standard filter (+0.15[-0.95 to 0.6]). The norepinephrine infusion rate was reduced during oXiris CRRT, but not during standard filter CRRT. In the second filter treatment period, there was no significant reduction in endotoxin or cytokine levels in either group.

**Conclusions:**

CRRT with the oXiris filter seemed to allow effective removal of endotoxin and TNF-α, IL-6, IL-8 and IFNγ in patients with septic shock-associated acute renal failure. This may be associated with beneficial hemodynamic effects.

## Background

Sepsis must always be treated with appropriate antibiotics and the focus of infection eradicated [1], but mortality rates remain high and additional therapies are required to improve outcomes. Lipopolysaccharide (LPS, endotoxin), a product of the outer Gram-negative bacterial wall, is an important bacterial toxin that can induce an immunologic response, involving the release of cytokines and other mediators [2–5] and causing vasodilation, endothelial leakage and organ dysfunction [6–8]. Circulating endotoxin can also be found in Gram-positive infections, probably originating by translocation from an ischemic gut [9]. One of several pathogen-associated molecular patterns, endotoxin is one of the principal triggers of septic shock occurring as a result of Gram-negative infection. Blood endotoxin levels have been correlated with the severity of illness, maximum organ dysfunction attained during the intensive care unit (ICU) stay and mortality [6, 8, 10]. Various therapeutic strategies have been used to attempt to neutralize the pathogenic activity of endotoxin, but with limited success [11–15]. The associated excessive cytokine response maintains and worsens septic shock and is associated with worse outcomes [16]. Specific therapies have also been developed to remove circulating cytokines [17].

The AN69-based oXiris membrane is modified with a positively charged poly-imine ethylene layer capable of adsorbing negatively charged endotoxin molecules. The adsorption of endotoxin has been demonstrated and quantified in *in vitro* settings [16, 18], but never tested in septic patients. In addition to its adsorption capability, the oXiris filter is otherwise an ordinary dialysis filter capable of providing a full dialysis treatment. To evaluate the efficacy of the oXiris filter in removing endotoxin and cytokines, we randomized, using a double-blind cross-over design, patients with septic shock-associated acute renal failure to receive continuous renal replacement therapy (CRRT) with either an oXiris filter or a standard filter. We hypothesized that the oXiris filter would remove endotoxin.

## Methods

### Study cohort

Over a two-year period (Feb 2016—Feb 2018), all consecutive adult patients on the ICUs at Skåne University Hospital in Malmö and in Lund and at Helsingborg Regional Hospital who had septic shock, with a blood culture positive for a Gram-negative bacteria or suspected to be caused by a Gram-negative agent, and associated Kidney Disease: Improving Global Outcomes (KDIGO) stage 3 acute renal failure [19], were considered for inclusion. Exclusion criteria were: age <18 years and/or known human immunodeficiency virus (HIV) or hepatitis B/C infection. The study was approved by the Regional Medical Research Ethics Board of Southern Sweden (Id 5/2013) and written informed consent was received from all patients or their next of kin. The study was registered at Clinicaltrials.gov (Id NCT02600312).

When a patient met the inclusion criteria, the physician-in-charge alerted the whole study team via an encrypted chat group on social media and an endotoxin pretest was performed on each patient to assess blood endotoxin load. Patients with a plasma endotoxin level >0.03 EU/ml were enrolled.

To test logistics before the study started, the protocol was used in two run-in patients who fulfilled inclusion criteria, had initial endotoxin levels >0.03 EU/ml and were randomized before start of treatment. Data from these patients were included before statistical analysis to increase the size of the cohort for the endotoxin evaluation but no cytokine analyses were carried out.

### Randomization, CRRT treatment and blinding of study filters

A cross-over regime consisting of 24 h treatment with an oXiris filter followed by 24 h treatment with a standard filter or the reverse filter order was used. The order of the two filters was randomly assigned using simple sealed opaque envelopes. oXiris and the standard filter used (Baxter M150ST) have the same filter housing, a volume of 189 ml, similar rheological parameters and were run on a Prismaflex machine using settings selected at the discretion of the attending physician. Both filters contain AN69, which can remove small amounts of endotoxin, but the oXiris filter has an additional superficial poly-imine ethylene layer with greater endotoxin adsorption capacity. The treating physician and staff as well as the patient were blinded to the type of filter by covering the brand marks on the front and the bar code on the back using non-transparent tape. At the end of the 1st filter period, a separate Prismaflex machine was primed with the new filter and the patient changed to the new machine. To be included in the analysis, patients need to receive interruption-free CRRT for the complete 1st treatment period and for at least the first 8 hours of the 2nd treatment period, enabling a minimum of 3 samples to be collected during the 2nd period.

### Measurements

The following clinical variables were recorded: main diagnosis prompting ICU admission, main comorbid diagnoses, simplified acute physiology (SAPS) 3 score at admission to the ICU and sequential organ failure assessment (SOFA) score on the two study days.

Blood samples were collected from an arterial line into a heparinized glass vial and a plastic vial coated with ethylene-diamine-tetra-acetic acid (EDTA) immediately before (0 h) and 1, 3, 8, 16 and 24 hours after the start of each filter period. Blood samples were immediately centrifuged and plasma was kept at minus 80°C until analysis. Samples for routine blood biochemistry analysis were drawn at the start of each filter period and at the end of the study.

Blood endotoxin concentrations were measured using assays based on *Limulus* amoebocyte lysate (LAL) [20, 21]. Heparinized plasma was diluted 1:10 in endotoxin-free cell culture grade water containing 0.02% Triton X-100 and heated to 70°C for 10 min. After cooling to room temperature, an equal volume of LAL reagent (Wako Limulus ES II Test) was added and the turbidity that developed during intermittent stirring at 37°C was measured on a microplate reader. The readout, i.e., time to an 8% decrease in transmittance at 405 nm, was compared to a standard curve constructed by analysis of healthy plasma with known concentrations of endotoxin. The lower detection limit in plasma was 0.03 EU/ml, corresponding to 4 pg/ml. All lab-ware used was either glass or glass-coated, including the microplates (Thermo Scientific WebSeal Plate+).

The endotoxin pretest was performed as previously described but with only two controls prepared from healthy plasma; one negative control without endotoxin and another with added endotoxin corresponding to 0.03 EU/ml in plasma. Results from the endotoxin pretests were obtained within two hours from blood sampling.

Cytokine levels were measured on EDTA plasma using the Bio-Plex Multiplex Immunoassay (Bio-Rad) according to the manufacturer’s instructions. The lower detection limits were (pg/ml): interleukin (IL)-1β, 0.31; IL-2, 1.0; IL-4, 0.09; IL-6, 1.0; IL-8, 1.2; IL-10, 2.0; granulocyte-macrophage colony-stimulating factor (GM-CSF) 0.7; interferon (IFN)γ, 0.28; tumor necrosis factor (TNF)-α, 3.4. Mean arterial pressure was measured continuously and norepinephrine infusion rate was measured hourly. Lactate levels were measured together with the endotoxin/cytokine blood sampling.

### Statistical analysis

Sample size calculation was based on changes in mean endotoxin level detected using the endotoxin activity assay (EAA) test in a previous study on extracorporeal endotoxin removal in patients with septic shock [22]. Using a Student's paired t-test with a two-sided α = 0.05, it was calculated that 80% power would be obtained with a sample size of 13 patients using a cross-over design, based on a decrease in endotoxin levels from 0.55 [0.44–0.68] EU/ml before treatment to 0.25 [0.13–0.41] EU/ml after treatment [22] with a least detectable difference with the LAL test of 0.03 EU/ml. To compensate for potentially larger variation in endotoxin levels, we estimated that 16 patients with complete datasets should be included.

Differences in absolute concentrations of endotoxin and cytokines at the different time points compared to baseline level were analyzed using ANOVA followed by the Wilcoxon signed rank test. Differences in baseline parameters were evaluated using a Mann-Whitney U-test. Complete endotoxin concentration profiles were evaluated using a Fisher's exact test. The primary endpoint was change in plasma endotoxin level during treatment, and secondary endpoints were changes in cytokine levels. Normally distributed data are presented as means and standard deviations and non-normal data as medians and lower/upper quartiles. Significance level was set at <0.05. We used SAS Statistical Analysis Software (Cary, NC, USA) for all calculations.

## Results

### Patients

**Fig 1:**
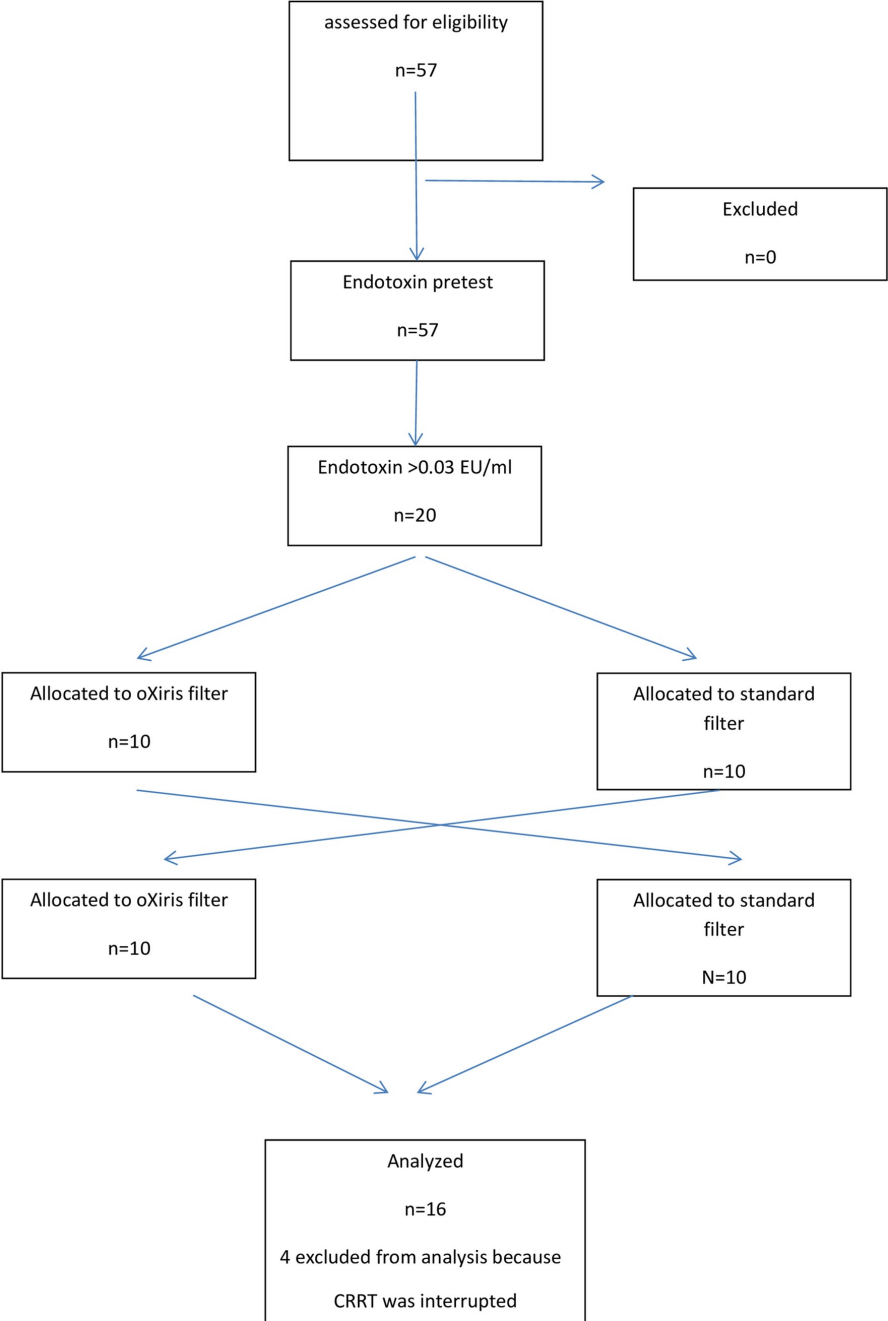
Consort diagram of the cohort.

Fifty-seven patients were screened and all met the inclusion criteria and underwent an endotoxin pre-test; 20 had an endotoxin level >0.03 EU/ml and were randomized (Fig 1). All patients were treated using continuous venovenous hemodiafiltration (CVVHDF) with regional citrate anticoagulation. Four patients did not complete the study regime because of prolonged pauses or termination of the CRRT treatment, and 16 patients were eventually studied. Demographic data of the cohort are presented in Table 1.

**Table 1:** Demographic data of the included patients.

Study patient Study center	Sex Age (years)	Source of infection	Bacterial growth in blood	SAPS3 score SOFA day1 SOFA day2	Filter sequence
**1** Malmö	male 75	cholangitis	*Escherichia coli* (ESBL)	62 12 8	o*X*iris-standard
**2** Malmö	female 80	perforated duodenum	none	77 10 12	Standard-o*X*iris
**3** Lund	female 74	unknown	none	63 15 16	Standard-o*X*iris
**4** Lund	male 61	cholangitis	none	67 16 14	o*X*iris-standard
**5** Helsingborg	male 73	cholecystitis	*E*. *coli*	77 16 16	o*X*iris-standard
**6** Lund	male 53	cholecystitis	*E*. *coli*	96 19 17	o*X*iris-standard
**7** Lund	female 80	unknown	none	81 9 6	Standard-o*X*iris
**8** Malmö	male 75	cholangitis, pancreatitis	*E*. *coli*	67 12 10	Standard-o*X*iris
**9** Malmö	male 70	unknown	*E*. *coli*	81 16 15	Standard-o*X*iris
**10** Lund	male 54	colon perforation	*Klebsiella oxytoca*	78 12 10	Standard-o*X*iris
**11** Malmö	male 66	ruptured urine bladder, previously reconstructed	*Pseudomonas aeruginosa*	48 missing missing	o*X*iris-standard
**12** Malmö	male 79	intestinal perforation	none	69 15 16	o*X*iris-standard
**13** Malmö	female 47	unknown	*E*. *coli*	83 18 14	Standard-o*X*iris
**14** Malmö	female 61	necrotizing gingivitis	*Pseudomonas aeruginosa*	79 9 10	Standard-o*X*iris
**15** Lund	female 81	cholangitis	*E*. *coli* (ESBL)	86 15 17	o*X*iris-standard
**16** Malmö	male 82	colon perforation	none	67 13 13	o*X*iris-standard
**run in A** Lund	female 72	pyelonephritis	*E*. *coli*	81 10 13	o*X*iris-standard
**run in B** Lund	female 76	pneumonia	none	112 16 14	o*X*iris-standard

ESBL: extended spectrum beta-lactamase

### First treatment period (0–24 hours)

Baseline laboratory and renal replacement therapy data are given in Table 2.

**Table 2:** Baseline data of the included patients before start of the 1^st^ treatment period.

Parameter	o*X*iris mean ± SD	ST mean ± SD	*P*-value
Creatinine, mmol/l	228 ± 49	316 ± 187	0.71
Urea, mmol/l	14.0 ± 3.1	20.8 ± 9.5	0.15
Hemoglobin, g/l	102 ± 7.1	97 ± 19.4	0.05
Hematocrit	0.3 ± 0.0	0.3 ± 0.1	0.03
Base excess, mmol/l	-6.2 ± 4.0	-5.3 ± 2.7	0.95
Potassium, mmol/l	3.9 ± 0.7	4.5 ± 1.0	0.16
Lactate, mmol/l	3.9 ± 2.6	2.2 ± 1.0	0.24
Leukocyte count x10^6^	23.4 ± 23.9	20.8 ± 12.1	0.96
CRP, mg/l	276 ± 63	269 ± 96	0.64
Norepinephrine infusion, μ/kg/min	0.48 ± 0.31	0.18 ± 0.12	0.02
CRRT blood flow	132 ± 69	121 ± 38	0.47
CRRT effluent flow	2634 ± 1185	2541 ± 1021	0.75

CRRT: continuous renal replacement therapy; CRP: C-reactive protein

#### Endotoxin levels

**Fig 2:**
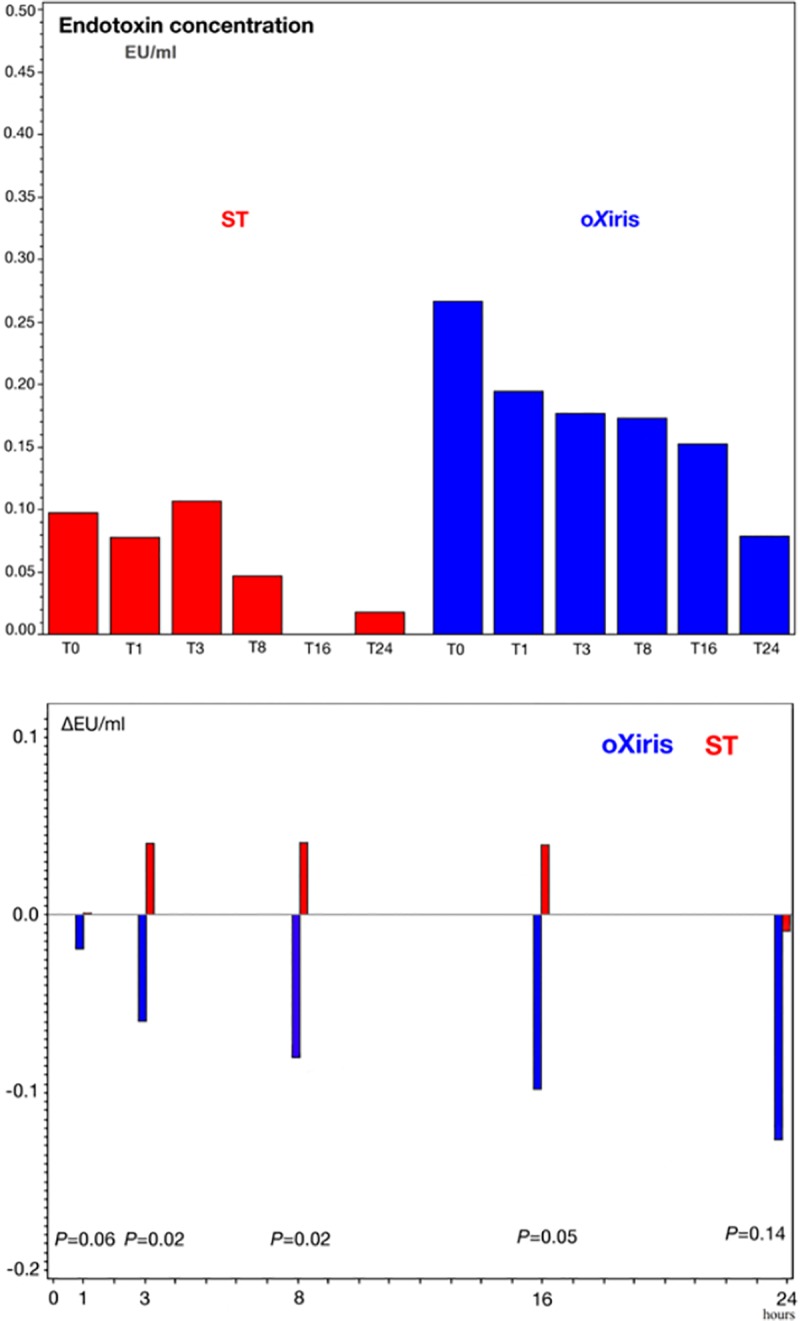
Endotoxin concentrations (EU/ml) during first filter treatment period (0–24 hours) in the standard (ST) and oXiris filter groups. Panel (a) shows the median endotoxin concentrations at each time point and Panel (b) shows the absolute mean change in endotoxin level.

The median baseline plasma endotoxin level [interquartile range] at T0 was 0.27 [0.15–0.63] EU/ml in the oXiris group (*n* = 8) and 0.10 [0.03–0.16] EU/ml in the standard filter group (*n* = 8, *P* = 0.06) (Fig 2). The endotoxin concentration decreased significantly more in the oXiris group at 3, 8, and 16 hours than in the standard filter group (*P* = 0.02, 0.02 and 0.05, respectively) (Fig 2).

Endotoxin was present in the blood in at least two sampling points in 7 oXiris and 6 standard filter patients. In order to increase power, endotoxin data from the two run-in patients, both randomized to oXiris during the first treatment period, were included for endotoxin profile comparison. For these 15 patients, we compared the endotoxin profiles in the 1^st^ treatment period. Endotoxin levels decreased during treatment in 7 of the 9 (77.8%) oXiris filter patients, but only in 1 of the 6 (16.7%) standard filter patients (*P* = 0.02).

#### Cytokine levels

**Fig 3:**
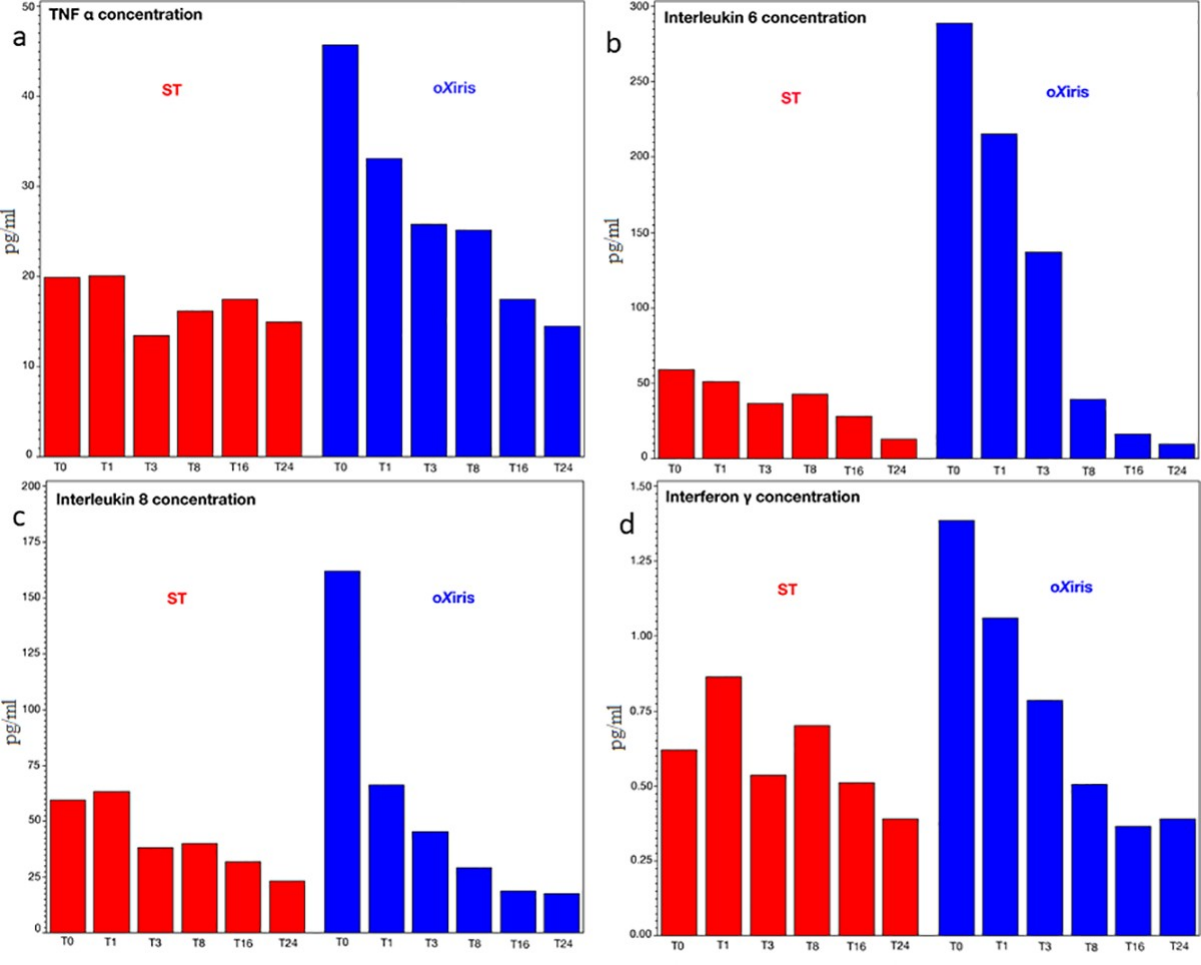
Blood cytokine levels during first filter treatment period (0–24 h) in the standard (ST) and oXiris filter groups. (a) tumor necrosis factor (TNF) α concentrations, (b) interleukin (IL)-6, (c) IL-8 and (d) interferon (IFN)γ.

TNF-α concentrations decreased more rapidly and to a greater degree (by about 70%) in the oXiris group compared to the standard filter group (by about 20%, *P* = 0.008) (Fig 3A). IL-6 and IL-8 levels decreased significantly compared to baseline (*P* = 0.008) during the 24-hour period for both filters, although in the standard filter group IL-6 values increased between T3 and T8 and IL-8 values increased between T0 and T1 and T3 and T8 (Fig 3B and 3C). IFNγ levels were just above the lower detection limit of the assay and decreased from the start of CRRT in the oXiris group and somewhat later in the standard filter group (Fig 3D).

Plasma levels of IL-1β, IL-2, IL-4, IL-10 and GM-CSF scarcely reached the lower detection limit and were not analyzed further.

#### Hemodynamic variables

**Fig 4:**
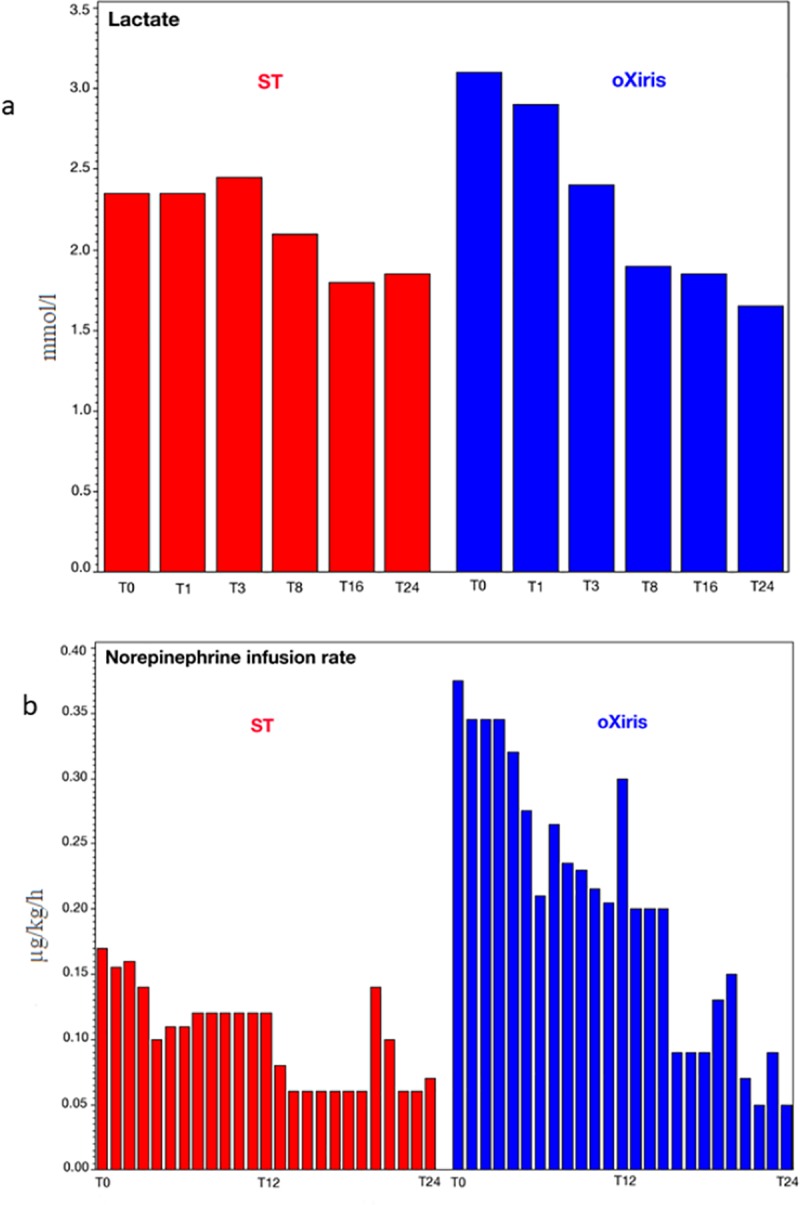
Median lactate levels (a) and norepinephrine doses (b) during first filter treatment period in the standard (ST) and oXiris filter groups (0–24 h).

At baseline, the median lactate concentration was 3.1 [2.2–6.1] mmol/L in the oXiris group and 2.4 [1.4–3.0] mmol/L in the standard filter group (*P* = 0.24). Blood lactate levels decreased by 1.3 [2.2–1.1] mmol/L in the oXiris group (*P* = 0.02), but remained essentially unchanged in the standard filter group +0.2 [-1.0 - +0.6] (Fig 4A).

Mean arterial pressure was maintained > 65 mmHg in both groups throughout the filter treatments (for the first treatment period, the median values were 69.5 [66–76.5] and 69.5 [57–74] (*P* = 0.75) for oXiris and standard filter groups, respectively). The median norepinephrine infusion rate at T0 was 0.38 [0.29–0.68] μ/kg/h in the oXiris group and 0.17 [0.11–0.24] μ/kg/h in the standard filter group (*P* = 0.02). In the oXiris group, there was a significant reduction in norepinephrine dose over the first 4 hours (T4, P<0.03), which continued throughout the 24-hour period; the median change during the 24-hour period was -0.25 [-0.57 - -0.17] μ/kg/h (*P* = 0.02); there was no significant decrease in the standard filter group -0.07 [-0.28–0.10] μ/kg/h (*P* = 0.38) (Fig 4B).

### Second treatment period (24–48 hours)

There was no significant change in endotoxin or cytokine levels in either group during treatment period 2 (S1 and S2 Figs). There was also no change in lactate concentrations or in norepinephrine infusion rate (S3 Fig).

## Discussion

The aim of this study was to evaluate the ability of the oXiris dialysis filter membrane to reduce endotoxin and cytokine levels during a 24-hour treatment period in patients with septic shock, and to compare the results with those obtained using a standard filter. Endotoxin levels decreased significantly using the oXiris filter compared to the standard filter. No further reduction in endotoxin levels occurred during the 2^nd^ treatment period in the cross-over setting.

Just over one third of the patients with septic shock who were screened had a positive endotoxin pretest based on an updated endotoxin LAL assay, which we believe is the most accurate method for quantifying the presence of endotoxin in plasma. Patients in the group in which oXiris was used during the 1^st^ treatment period seemed, despite the randomization process, to be more severely ill, as indicated by somewhat higher baseline concentrations of endotoxin and cytokines, higher lactate levels and higher doses of norepinephrine.

Endotoxins promote the secretion of pro-inflammatory cytokines and can induce a shock state in humans [3]. The endotoxin load can be a short initial pulse, which is quickly cleared by the immune system, mainly by the liver, or it may be the result of an imbalance between the clearance capacity of an overwhelmed immune system and constant production from the infecting bacteria. The degree of encapsulation of the infectious foci, size of the bacterial colony, the specific bacterial species and the effectiveness of any antibiotic therapy can all contribute to the endotoxin load [6,7]. Any endotoxin not cleared by the immune system is available for adsorption when blood is pumped through a dialyzer with an appropriate filter. Theoretically, blood flow through the filter, concentration of endotoxin, capacity of the membrane to adsorb the endotoxin molecules and total surface of the filter are the most important factors governing the endotoxin reduction rate [2–5]. After a period of time, the membrane will become saturated and the filter must be changed.

Several options to remove endotoxins from the blood have been tested. High flow hemofiltration and hemodialysis do not seem to work [23]. Studies with the endotoxin binder, polymyxin B, immobilized on polystyrene fibers (PMX), able to eliminate endotoxin from the blood pumped through the circuit, have mainly been negative. In the recent North American EUPHRATES study, use of the PMX filter in patients with septic shock was not associated with improved survival compared to standard treatment [24]. An interesting finding in our study was that the endotoxin levels remained elevated despite ongoing CRRT treatment. A treatment regime with intermittent endotoxin removal just a few hours per day, as performed in the EUPHRATES trial, may therefore be insufficient to demonstrate an impact on outcomes, potentially explaining the negative results.

The CytoSorb adsorber is composed of biocompatible porous polymer beads that remove substances from whole blood based upon pore capture and surface adsorption. Larger blood components, such as blood cells, pass the beads without entering the pores. Molecules of size 5–60 kDa, such as cytokines, bilirubin, myoglobin, drugs and some metals, are adsorbed. Endotoxin is, however, not very well adsorbed. The CytoSorb adsorber can be used as a standalone treatment, or combined in tandem with CRRT. Large multicenter studies using this filter are lacking at the present time [16]. In *in vitro* studies, the oXiris filter was the only hemoperfusion device tested that showed both endotoxin and cytokine removal [18].

Cytokines are known to mediate the host response to infection, but their excessive release can contribute to organ damage and reduction in circulating levels may therefore be beneficial [17]. Importantly, indiscriminate removal of all cytokines may impair immune regulation; however, when one or more cytokines is present in excess, as during sepsis, the proportion removed by adsorption will be greater than that of cytokines present at lower concentrations, thus, in theory, helping restore cytokine balance [25]. Circulating levels of TNF-α, IL-6, IL-8 and IFNγ were significantly lowered by both filters, but to a greater extent with oXiris than with the standard filter. The rest of the cytokines analyzed (IL-1β, IL-2, IL-4, IL-10 and GM-CSF) were present in very low concentrations and were not compared across groups.

TNF-α is excreted by leukocytes and macrophages following recognition of an antigen (endotoxin). Its main function is to recruit additional leukocytes and monocytes in the blood and to increase the inflammatory process by increasing adhesion to the endothelium and secretion of molecules belonging to the complement system by the liver [2–5]. IL-6 and IL-8 are mediators of the acute phase response in infection. IFNγ is mainly secreted by T lymphocytes after antigen (endotoxin) stimulation and has numerous effects, including promotes differentiation of T lymphocytes and natural killer cell activity [2–5].

Blood lactate concentrations decreased significantly more in the oXiris group during the first 24-hour treatment period, and the doses of norepinephrine were also reduced after just 4 hours in the oXiris group. These observations suggest that the endotoxin and cytokine removal properties of the oXiris filter may contribute to improve hemodynamic status. However, this hypothesis needs to be explored further in larger studies.

The main limitation of this study is the small cohort size consisting of only 16 patients. The prospective double-blinded regime is laborious and cumbersome and the analyses are expensive and time-consuming. Therefore, we could not enlarge the cohort at the present time. The small cohort also influenced our choice of statistical test. Indeed, a parametric mixed model for repeated tests gave a non-significant difference between groups, but a simulation showed that 30 subjects would have been needed in each group for optimal input conditions. We therefore chose to report the results of non-parametric testing, although we acknowledge that this weakens the interpretation of our results. A second limitation is that no further reduction of endotoxin or cytokines occurred during the 2^nd^ treatment period because all levels were already low at the start of the second session. This may indicate that an equilibrium of endotoxin had been reached between the filter membrane and the blood compartment, suggesting that the affinity for endotoxin is somewhere between 0.01 and 0.03 EU/ml. Finally, by randomization bias, the patients who were randomized to oXiris as the first filter were more ill than those randomized to standard filter first, with higher endotoxin and cytokine levels, and it could be argued that this may limit the conclusions that can be drawn from our results. However, our primary goal was to show that the oXiris filter removes endotoxins and cytokines in a clinical setting, and not to compare it with the standard filter, and we have undoubtedly found a signal for this in the cohort. Both filters possess an AN69 layer with limited adsorbing capacity; the standard filter group was therefore not a true zero control.

In conclusion, using a patient selection process based on an endotoxin pretest, CRRT treatment with an oXiris filter showed a strong signal of a reduction in circulating endotoxin and cytokine levels. This reduction seemed to be associated with a favorable hemodynamic effect, as suggested by a more rapid decrease in blood lactate levels and lower doses of norepinephrine needed to maintain the mean arterial pressure. There was a blunted cytokine response in both filter groups, but the reduction in TNF-α, IL-6, IL-8 and IFNγ was more substantial in the oXiris than in the standard filter group.

## Supporting information

S1 Fig Median endotoxin concentrations (EU/ml) during second filter treatment period in the standard (ST) and oXiris filter groups. (TIF)

S2 Fig Median cytokine concentrations (pg/ml) during second filter treatment period in the in the standard (ST) and oXiris filter groups. (a) tumor necrosis factor (TNF) α concentrations, (b) interleukin (IL)-6, (c) IL-8 and (d) interferon (IFN)γ.
(TIF)

S3 Fig Median lactate (mmol/l) levels (a) and norepinephrine (μg/kg/h) doses (b) during second filter treatment period in the standard (ST) and oXiris filter groups. (TIF)

S1 File oXiris protocol. (PDF)

S2 File CONSORT checklist. (PDF)
