# Supplementary figures and images for: Endotoxin and cytokine reducing properties of the oXiris membrane in patients with septic shock: A randomized crossover double-blind study
Source: PLoS One. 2019 Aug 1;14(8):e0220444. doi: 10.1371/journal.pone.0220444 (PMC6675097; doi:10.1371/journal.pone.0220444)

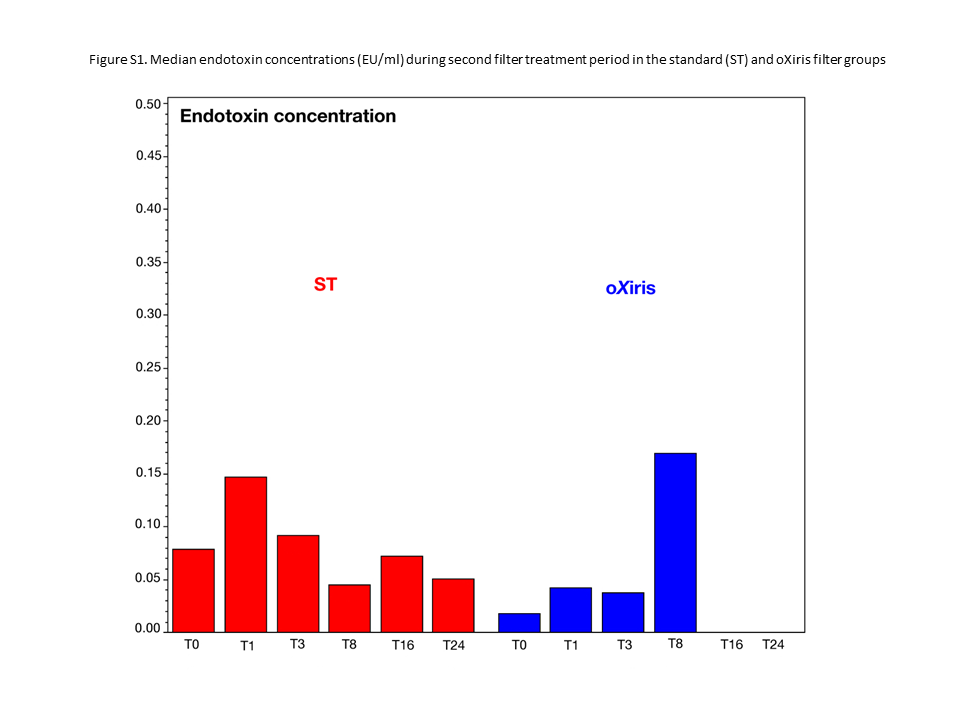

Supplement: S1 Fig — (TIF) [file pone.0220444.s001.tif]

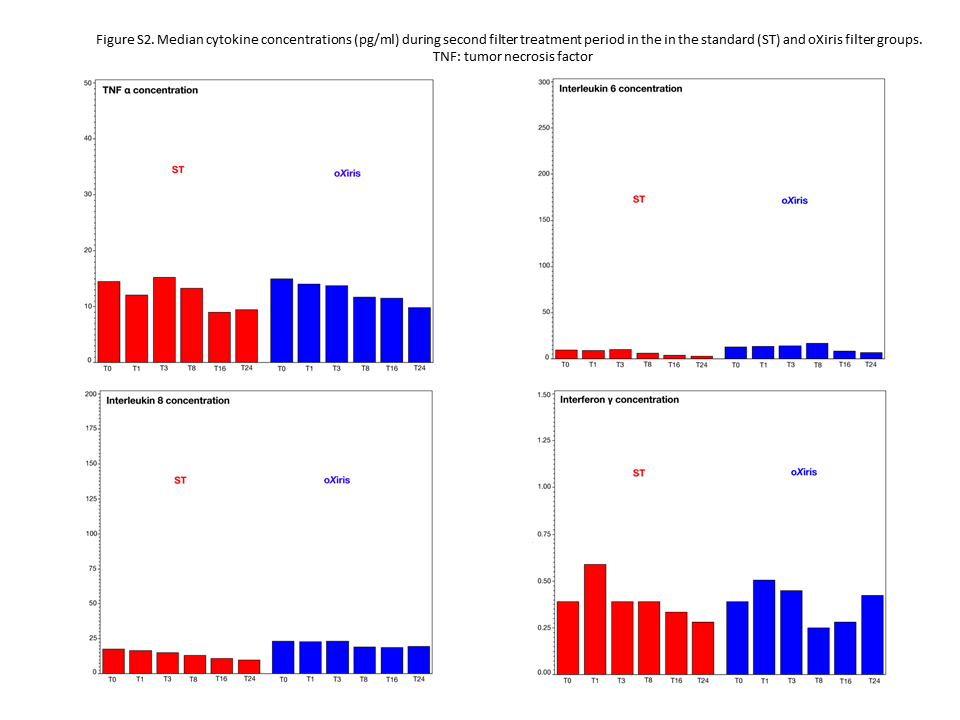

Supplement: S2 Fig — (a) tumor necrosis factor (TNF) α concentrations, (b) interleukin (IL)-6, (c) IL-8 and (d) interferon (IFN)γ. (TIF) [file pone.0220444.s002.tif]

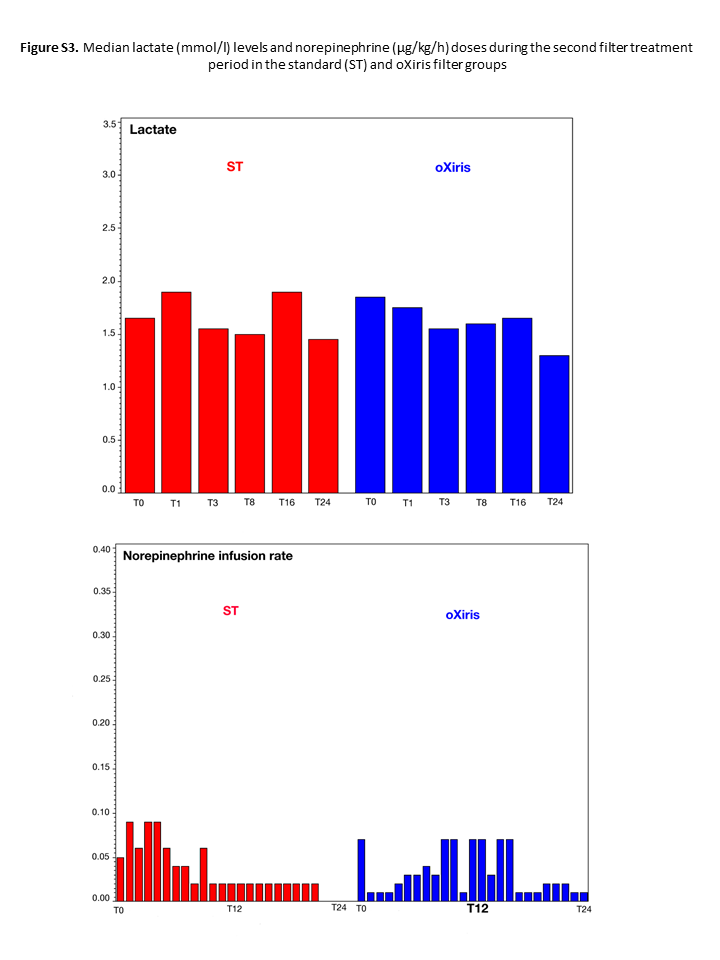

Supplement: S3 Fig — (TIF) [file pone.0220444.s003.tif]
