# Supplementary material for: Endotoxin and cytokine reducing properties of the oXiris membrane in patients with septic shock: A randomized crossover double-blind study
Source: PLoS One. 2019 Aug 1;14(8):e0220444. doi: 10.1371/journal.pone.0220444 (PMC6675097; doi:10.1371/journal.pone.0220444)
Supplement: S1 File — (PDF) [file pone.0220444.s004.pdf]

RESEARCH PROTOCOL

Date: 7 June 2015
Version: Final Amended II

Continuous renal replacement therapy with the endotoxin and cytokine adsorbing filter, Oxiris®, in patients with severe sepsis. Effects on circulating endotoxin, cytokine levels, respiration and the cardiovascular system in a randomized clinical cross-over study.

Titel in Swedish: Kan behandling med endotoxinadsorberande dialysfilter lindra sjukdomsförloppet och minska mortaliteten hos patienter med allvarlig sepsis eller septisk chock?

Ingrid Berkestedt, MD, PhD, Attending Specialist Anaesthesiologist, ingrid.berkestedt@med.lu.se
Marcus Broman, MD, PhD, Attending Specialist Anaesthesiologist/Intensivist, ewert.broman@telia.com
Mikael Bodelsson, MD, PhD, Professor of Anaesthesiology and Intensive Care, mikael.bodelsson@med.lu.se
Kristinn Örn Sverrisson, MD, Attending Specialist Anaesthesiologist/Intensivist, kristinn.sverrisson@med.lu.se

Department of Intensive and Perioperative Care, Skåne University Healthcare, SE-221 85 Lund, Sweden.

Purpose and aims

Defining the problem

Severe sepsis is an infection resulting in organ dysfunction including circulatory, respiratory and kidney failure. Untreated, the condition can progress into septic shock with a mortality of 30-60%. The septic reaction is triggered by bacterial substances, such as endotoxin, and is maintained by inflammatory mediators of the host e.g. cytokines. Acute kidney failure is a common feature of septic multi organ failure and the affected patients need support by continuous renal replacement therapy (CRRT) i.e. dialysis and/or haemofiltration. This opens up a possibility to dampen the septic reaction by simultaneously removing bacterial substances and cytokines extracorporally.

The Oxiris® filter is a registered product for CRRT already safely used in routine care. In *in vitro* experiments, the Oxiris® filter has been demonstrated to adsorb endotoxin and cytokines. Compared to conventional filters this may be advantageous in patients with severe sepsis but neither decreased levels of endotoxin and cytokines nor an improved outcome has been demonstrated with clinical use.

Purpose

The long term purpose of the studies outlined in the present research programme is to contribute to improving the outcome of patients with severe sepsis.

Specific aims

We want to determine if continuous renal replacement therapy with the Oxiris® filter is beneficial in patients with severe sepsis. Before outcome studies with overall end-points such as

mortality or complications to sepsis can be performed the following questions must be addressed:

- Does use of the Oxiris® filter in patients with severe sepsis decrease plasma levels of endotoxin and/or cytokines more than conventional filters?
- Does use of the Oxiris® filter in patients with severe sepsis improve hemodynamics compared to conventional filters?
- Does use of the Oxiris® filter in patients with severe sepsis improve respiratory function compared to conventional filters?

If the answer to one or more of these questions is Yes we will proceed addressing the question:

- Does use of the Oxiris® filter in patients with severe sepsis improve survival compared to conventional filters?

Survey of the field

Pathophysiology of sepsis

Sepsis, a complex clinical syndrome caused by an infection with bacteria, viruses or fungi, is triggered by microbial components such as endotoxin (lipopolysaccharide, LPS). The pathophysiology includes an overwhelming inflammatory host response, which can lead to the development of multiple organ failure, resulting in mortality rates of up to 45 % (1). Sepsis in conjunction with one or more failing organs is called severe sepsis. Numerous trials have been performed in order to improve outcome with anti-inflammatory agents blocking the action of inflammatory mediators released in sepsis e.g. cytokines such as tumor necrosis factor (TNF)-α and interleukin (IL)-1 (2, 3). Although promising in *in vivo* studies on experimental animals the results have been disappointing in the clinical setting (4). One reason could be that the antagonists disturb the regulation of the inflammatory response, thereby impairing the immunological actions directed towards the pathogens.

Blood purification

An attractive alternative approach is to reduce the levels of the agents triggering and maintaining the septic reaction. The overall concept of such blood purification is to attenuate the overwhelming systemic expression of pro- and anti-inflammatory mediators. Subsequent restoration of immune homeostasis is thought to be able to improve outcome and survival. Thus, the blood purification concept and therapies have evolved toward the non-specific removal of a broad spectrum of inflammatory mediators, which can also include microbial toxins (5). Extracorporeal removal of toxins and cytokines has been achieved using two different principles:

1. Extracorporeal filtering
2. Extracorporeal hemoadsorption

1. Extracorporeal filtering

Kidney failure is prevalent in sepsis making up severe sepsis. Around 5% of critically ill patients undergo continuous renal replacement therapy because of acute kidney injury. This therapy often includes dialysis but nowadays usually also haemofiltration, both modalities achieved with the same setup. In High-volume haemofiltration (HVHF) an increased filtration pressure and/or pore size of the filtration sieve allows filtration of larger molecules such as bacterial toxins and host-derived cytokines (5). A recent Cochrane report suggested that there are no adverse effects of HVHF. The authors concluded, however, that there is insufficient evidence to recommend the use of HVHF in critically ill patients with severe sepsis and or septic shock (6).

2. Extracorporeal hemoadsorption

This technique utilizes the more or less selective affinity between bacterial toxins/cytokines and resins attached to a membrane. Toxins and/or cytokines in blood flowing by the membrane are trapped and the blood is purified. This principle is employed by the Toraymyxin® cartridge for removal of endotoxin by binding to covalently bound polymyxin B resins in the cartridge. In a small randomized controlled trial of 64 septic shock patients published in JAMA 2009, Cruz and colleagues demonstrated a significantly improved organ function in the group treated with the cartridge (7). More importantly, 27-day mortality was significantly lower in the treatment group.
The Alteco® LPS Adsorber is a device working according to a similar principle. In a series of septic shock cases, it was observed that treatment with the adsorber was associated with a reduced need for pressor administration and organ function improved (8). No results from any study aimed at evaluating mortality have as yet been published.

The Oxiris® filter

Conventional filters for continuous dialysis/haemofiltration, such as AN 69 and AN 69 ST, binds low molecular weight polypeptides including some cytokines. Frequent change of filters every three hours to avoid saturation have been demonstrated to result in lower levels of IL-6, IL-10 and IL-18 as well as increased hemodynamic stability in patients with septic shock (9). The Oxiris filter is a further development displaying higher biocompatibility including less activation of the contact system and a heparin coating. In addition, the Oxiris® filter binds endotoxin and clearance of TNF-α, IL-1ra, IL-1β and IL-10 from human blood *in vitro* is greater than with AN 69 (10).

In an *in vivo* study on anaesthetized pigs challenged with live *Pseudomonas aeruginosa*, treatment with Oxiris® filter decreases circulating endotoxin and IL-1β levels and reduces need for fluids compared to treatment with AN 69 (10). Importantly, signs of metabolic acidosis as well as lactate levels were improved indicating reversal of the pseudomonas-induced septic shock. The decrease in endotoxin levels was, however, transient despite continuous use of the Oxiris® filter suggesting rapid saturation of its endotoxin-scavenging capability. This leaves an important issue to be dealt with: How often must the Oxiris® filter be changed in order to retain its adsorptive function?

Despite absence of clinical studies, the Oxiris® filter has gained widespread use and there have been no safety issues. Still, there is uncertainty weather the treatment is actually effective. The present project aims at relieving this uncertainty.

Project description

Theory

If we in a first study can demonstrate that use of the Orixis® filter in sepsis patients reduces endotoxin levels, cytokine levels or improves sepsis-induced failing circulation or respiration, this will be proof of concept of a second randomized controlled outcome study evaluating the effect of Oxiris® treatment on sepsis complications and mortality.

Study population

Patients >18 years, both genders, severe sepsis or septic shock, acute kidney failure.

Inclusion criteria

- Patients admitted to intensive care unit (ICU) of age >18 years with decision

based on the clinical situation of the patient taken by the physician in charge that continuous renal replacement therapy will be started

AND

- Vasoconstrictor and volume dependent septic shock with known Gram-negative infectious agent in blood culture

OR

- Vasoconstrictor and volume dependent septic shock suspected to be caused by a Gram-negative agent and with positive plasma endotoxin test

Exclusion criteria

- Infected with Hepatitis B or C or HIV
- Dependence on dialysis treatment before the actual ICU episode

Method

A randomized cross-over design will be used. Patients will be included after informed consent of patient or next of kin. Patients who recover will be informed again and if consent is not obtained the patient will be excluded. Eight patients will be randomized to CRRT with a conventional AN 69 M150 filter for 24 hours followed by Oxiris® filter for another 24 hours. Another eight patients will be randomized first to obtain treatment with Oxiris® filter for 24 hours followed by AN 69 M150 filter for another 24 hours.

Arterial blood samples will be drawn at start and then 1, 3, 8, 16 and 24 hours after the start of each filter, and analyzed for endotoxin (EAA assay), TNF-α, IL-1β, IL-6 and IL-10 (ELISA) levels. Standard blood tests will be analyzed simultaneously. Data concerning mode and settings of CRRT, heart rate, blood pressure, medication, data concerning ventilatory support and pathogen will be registered.

Power analysis based on a previous study on extracorporeal endotoxin removal in sepsis patients (8) suggested that if the material is analyzed with Student's paired t-test with $\alpha = 0.05$, a power above 0.8 will be obtained with 13 patients. To compensate for exclusion, 16 patients will be included. The cross-over design will compensate for any change in endotoxin and/or cytokine levels with time.

Time schedule

This first part of the study will be conducted at a single center and inclusion will start August 2015 and hopefully be completed 1½ year later. Results will be published within a year after completing the inclusion.

Statistics

Primary endpoint

Levels of endotoxin and cytokines will be compared using Student's paired t-test on AUC values for each 24-hour period. If the data does not follow a Gaussian distribution Wilcoxon signed-rank test will be used.

Changes over time will be analyzed using two-way repeated measures ANOVA.

Secondary endpoints

Please see primary endpoints.

Power analysis

Expected mean differences in endotoxin levels including standard deviation was based on data from a previous study on extracorporeal endotoxin removal in sepsis patients (reference: Ala-Kokko TI, Laurila J, Koskenkari J. Blood Purif.

2011; 32(4): 303-9). Power analysis suggested that if the material is analyzed with Student's paired t-test with $\alpha = 0.05$, a power above 0.8 will be obtained with 13 patients. To compensate for exclusion, 16 patients will be included. The cross-over design will compensate for any change in endotoxin and/or cytokine levels with time.

Ethics

Ethics Board Approval obtained by Regional Research Ethics Board in Lund, Sweden, 7th May 2013.

Adverse events reporting

The patients are located in an intensive care unit. oXiris filters are already at present included among the treatment options available to the physician in charge of the patient. During the study period there will always be one member (clinically experienced ICU physician) of the study personnel on call. If a suspicion arises of an Adverse Event this person will be called to the bedside to evaluate and participate in the scenario. In addition regular ICU staff will be present. This ensures that a report can be sent to Baxter within 24 hours.

Project organization

The study will be managed at the investigators' own department which harbors a suitable infrastructure including two intensive care units with established routines for CRRT (in Lund and Malmö, respectively), access to the help of a research nurse and well equipped laboratory facilities.

Dr. Ingrid Berkestedt is a skilled clinician with previous experience in clinical intensive care research including cytokine measurements in sepsis.

Dr. Marcus Broman is a renowned intensivist with previous scientific production within the field of CRRT.

Prof. Mikael Bodelsson is a specialist of inflammatory activation and endotoxin-related mechanisms in sepsis.

The project is investigator initiated.

Significance

Severe sepsis and sepsis with failing circulation that does not respond to fluid treatment, septic shock, is a major killer in Intensive Care Units with mortality rates ranging from 30 to 60 %. Despite increased knowledge of relevant pathophysiology, treatment is still restricted to antibiotics and support of failing vital organ function such as ventilator therapy. Recent results suggesting that extracorporeal scavenging of endotoxin can reduce mortality, at least in sepsis caused by Gram-negative bacteria, may prove to be a breakthrough (7). Establishment of the required extracorporeal circuit is, however, cumbersome in hospitals absent of cardiothoracic surgery.

CRRT is today standard of care for intensive care patients with failing kidney function. The Oxiris® membrane set up is a convenient alternative to conventional membranes for CRRT. The cytokine and endotoxin binding properties of the membrane surface suggests that it may be as effective as alternative extracorporeal endotoxin scavengers. Advantages include simultaneous CRRT, often needed in these patients, as well as a high level of patient safety due to established routines for bedside handling. This has made the Oxiris® membrane already a popular choice for CRRT in sepsis patients although several issues need to be addressed:

- there is uncertainty weather the treatment is actually effective in removing cytokines and/or endotoxin in patients
- there is uncertainty as to when the membrane gets saturated with cytokines and/or endotoxin calling for change to a fresh filter

These problems will be resolved by the outlined study generating information needed to in detail design the *second study* to answer the question:

- Does CRRT with Oxiris® filter reduce mortality of sepsis and/or reduce complications to sepsis in surviving patients?

Dissemination

A manuscript with the results of the study will be prepared and submitted to a scientific journal in the intensive care field within 6 month after the last patient has completed the 48-hour CRRT treatment, i.e. during the 2nd half of 2017. Meeting abstracts may be submitted with the same time schedule.

References

1. Hotchkiss, R. S., and I. E. Karl. 2003. The pathophysiology and treatment of sepsis. N. Engl. J. Med. 348:138-50.
2. Cohen, J. 2002. The immunopathogenesis of sepsis. Nature 420:885-91.

3. Tetta C, Bellomo R, Inguaggiato P, Wratten ML, Ronco C. Endotoxin and cytokine removal in sepsis. Ther Apher. 2002;6:109–115.

4. Deans KJ, Haley M, Natanson C, Eichacker PQ, Minneci PC. Novel therapies for sepsis: a review. J Trauma. 2005;58:867–874.

5. Rimmelé T, Kellum JA. Clinical review: Blood purification for sepsis. Crit Care 2011, 15, 205.

6. Borthwick EM, Hill CJ, Rabindranath KS, Maxwell AP, McAuley DF, Blackwood B. High-volume haemofiltration for sepsis. Cochrane Database Syst Rev. 2013 Jan 31;1:CD008075.doi: 10.1002/14651858.CD008075.pub2

7. Cruz DN, Antonelli M, Fumagalli R, Foltran F, Brienza N, Donati A, Malcangi V, Petrini F, Volta G, Bobbio Pallavicini FM, Rottoli F, Giunta F, Ronco C. Early use of polymyxin B hemoperfusion in abdominal septic shock: the EUPHAS randomized controlled trial. JAMA 2009;301:2445-2452.

8. Ala-Kokko TI, Laurila J, Koskenkari J. A new endotoxin adsorber in septic shock: observational case series. Blood Purif. 2011; 32(4): 303-9.

9. Haase M, Silvester W, Uchino S, Goldsmith D, Davenport P, Tipping P, Boyce N, Bellomo R. A pilot study of high-adsorption hemofiltration in human septic shock. Int J Artif Organs. 2007 Feb;30(2):108-17.

10. Rimmelé T, Assadi A, Cattenoz M, Desebbe O, Lambert C, Boselli E, Goudable J, Etienne J, Chassard D, Bricca G, Allaouchiche B. High-volume haemofiltration with a new haemofiltration membrane having enhanced adsorption properties in septic pigs. Nephrol Dial Transplant. 2009 Feb;24(2):421-7.
